# Machine learning-based integrated identification of predictive combined diagnostic biomarkers for endometriosis

**DOI:** 10.3389/fgene.2023.1290036

**Published:** 2023-11-27

**Authors:** Haolong Zhang, Haoling Zhang, Huadi Yang, Ahmad Naqib Shuid, Doblin Sandai, Xingbei Chen

**Affiliations:** ^1^ The First Affiliated Hospital of Zhejiang Chinese Medical University (Zhejiang Provincial Hospital of Chinese Medicine), Hangzhou, China; ^2^ Department of Biomedical Sciences, Advanced Medical and Dental Institute, Universiti Sains Malaysia, Penang, Malaysia; ^3^ Department of Community Health, Advanced Medical and Dental Institute, Universiti Sains Malaysia, Penang, Malaysia

**Keywords:** endometriosis, combined biomarkers, machine learning, predictive, diagnostic

## Abstract

**Background:** Endometriosis (EM) is a common gynecological condition in women of reproductive age, with diverse causes and a not yet fully understood pathogenesis. Traditional diagnostics rely on single diagnostic biomarkers and does not integrate a variety of different biomarkers. This study introduces multiple machine learning techniques, enhancing the accuracy of predictive models. A novel diagnostic approach that combines various biomarkers provides a new clinical perspective for improving the diagnostic efficiency of endometriosis, holding significant potential for clinical application.

**Methods:** In this study, GSE51981 was used as a test set, and 11 machine learning algorithms (Lasso, Stepglm, glmBoost, Support Vector Machine, Ridge, Enet, plsRglm, Random Forest, LDA, XGBoost, and NaiveBayes) were employed to construct 113 predictive models for endometriosis. The optimal model was determined based on the AUC values derived from various algorithms. These genes were then evaluated using nine machine learning algorithms (Random Forest, SVM, Gradient Boosting Machine, LASSO, XGB, NNET, Generalized Linear Model, KNN, and Decision Tree) to assess significance scores and identify diagnostic genes for each algorithm. The diagnostic value of these genes was further validated in external datasets from GSE7305, GSE11691, and GSE120103.

**Results:** Analysis of the GSE51981 dataset revealed 62 DEGs. The Stepglm [Both] and plsRglm algorithms identified 30 genes with the most potential using the AUC evaluation. Subsequently, nine machine learning algorithms were applied to select diagnostic genes, leading to the identification of five key diagnostic genes using the LASSO algorithm. The ADAT1 gene exhibited the best single-gene predictive performance, with an AUC of 0.785. A combination of genes (FOS, EPHX1, DLGAP5, PCSK5, and ADAT1) achieves an AUC of 0.836 in the test dataset. Moreover, these genes consistently exhibited an AUC exceeding 0.78 in all validation datasets, demonstrating superior predictive performance. Furthermore, correlation analysis with immune infiltration strengthened their predictive value by demonstrating the close relationship of the diagnostic genes with immune infiltrating cells.

**Conclusion:** A combination of biomarkers consisting of FOS, EPHX1, DLGAP5, PCSK5, and ADAT1 can serve as a diagnostic tool for endometriosis, enhancing diagnostic efficiency. The association of these genes with immune infiltrating cells reveals their potential role in the pathogenesis of endometriosis, providing new insights for early detection and treatment.

## 1 Introduction

In the rapidly evolving landscape of modern medical technology, precision medicine has spearheaded a revolutionary transformation in healthcare, offering patients tailored and customized medical services. Profound shifts are taking place within the medical community, particularly in areas such as disease diagnosis, prognosis, and treatment methodologies. Amidst these transformative changes, endometriosis has emerged as a compelling focal point worthy of attention. Endometriosis, a common gynecological disorder, refers to the abnormal growth of endometrial-like tissue outside the uterus, often manifesting as dysmenorrhea, chronic pelvic pain, infertility, and dyspareunia (Leyland et al., 2010). It is estimated that 2%–10% of women of reproductive age worldwide are affected by endometriosis, with 5%–21% experiencing severe pelvic pain. In infertile women, the proportion can reach up to 50%, associated with a 50% increase in the risk of ovarian cancer (Dunselman et al., 2014; Vercellini et al., 2014). This condition significantly impacts many women’s quality of life and reproductive health.

The diagnosis of endometriosis includes detailed symptom inquiry, clinical examination, and imaging techniques such as ultrasound and MRI to observe the ectopic tissues and cysts within the pelvic region (Smolarz et al., 2021). Laparoscopic surgery is used for direct visualization of ectopic tissues, combined with pathological examination to confirm the diagnosis (Koninckx et al., 2021). Treatment options include nonsteroidal anti-inflammatory drugs and hormone therapy to relieve pain and control the condition. Surgical treatments involve laparoscopic surgery to remove ectopic tissues and improve symptoms (Chapron et al., 2019; Kalaitzopoulos et al., 2021). Individualized treatment plans consider the severity, age, and fertility requirements to ensure the best outcomes. Early comprehensive treatment can alleviate symptoms, improve quality of life, and benefit overall health and fertility prospects.

However, despite the gradual progress of research into this disease, numerous mysteries remain concerning its etiology and pathogenesis, posing challenges to clinical diagnosis and treatment. Biomarkers, as indicators of specific molecules or features within the body, offer valuable insights into disease prediction, diagnosis, and treatment. Nevertheless, due to the complexity and diversity of endometriosis, the application of a single biomarker is limited, necessitating more comprehensive approaches to aid in disease identification, prediction, and diagnosis.

Machine learning is a powerful data analysis tool, which enhances predictive performance and stability by integrating multiple models, offering a fresh perspective for predicting combined biomarkers related to endometriosis. By amalgamating information from various biomarkers, machine learning ensemble methods can discern potential diagnostic combinations of strongly-associated biomarkers with the disease, providing robust support for developing precise diagnostics and personalized treatment strategies.

Therefore, the objective of this study is to utilize a comprehensive machine learning approach to identify combined biomarkers associated with the diagnosis of endometriosis, thereby enhancing the precision of prediction and diagnosis. By integrating diverse biological information, the aim is to identify key diagnostic biomarker combinations that hold significant predictive value, offering more effective support for early prediction and diagnosis of endometriosis.

## 2 Materials and methods

### 2.1 Data sources

**FIGURE 1:**
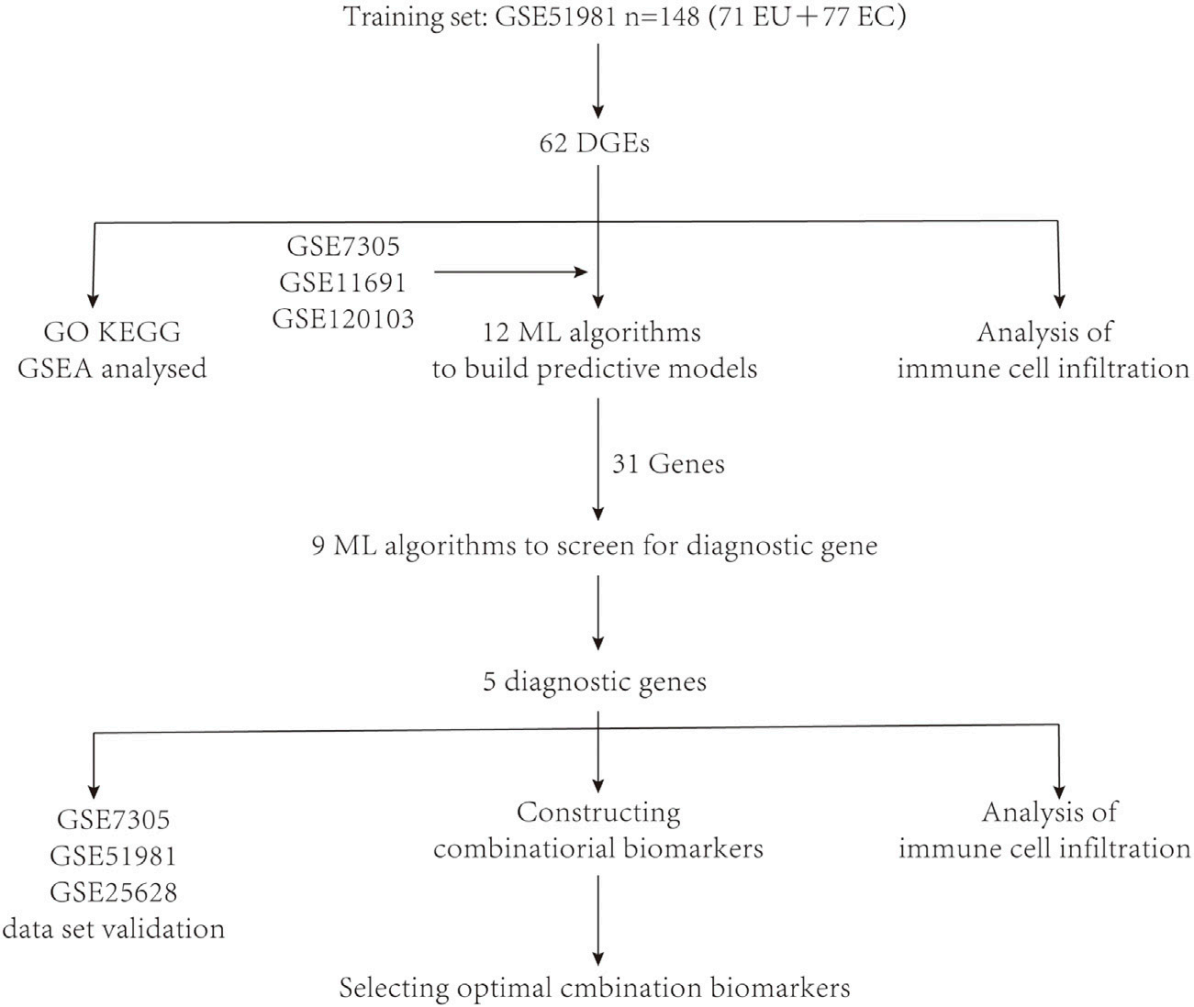
A schematic representation of the research design was created.

Four datasets were downloaded from the Gene Expression Omnibus (GEO) database https://www.ncbi.nlm.nih.gov/geo/,: GSE51981, GSE7305, GSE11691, and GSE120103. Profiles of four datasets are listed in Table 1. During the data preparation phase, Perl (version 5.30) scripts performed ID conversion on these datasets and computed the average values for duplicated gene names. Subsequently, the limma package (version 4.3.0) was employed within the R environment to conduct a differential analysis on the data. In the process of differential analysis, filtering criteria are set to retain only those differentially expressed genes that satisfied the conditions (| log2FC |) > 1 and *p* < 0.05. Additionally, a schematic representation of the research design was created (Figure 1).

**TABLE 1:** Sample information for transcriptome data.

	GEO name	Sample size	Control samples	Patient samples	Platforms	Published	References
Training Set	GSE51981	148	77	71	GPL570	01 Oct 2014	Tamaresis et al. (2014)
Test Set	GSE7305	20	10	10	GPL570	09 Apr 2007	Hever et al. (2007)
	GSE11691	18	9	9	GPL96	22 Dec 2008	Hull et al. (2008)
	GSE120103	36	18	18	GPL6480	26 Feb 2019	Bhat et al. (2019)

### 2.2 Construct predictive models

A repertoire of 11 classical algorithms, encompassing Lasso, Stepglm, glmBoost, SVM, Ridge, Enet, plsRglm, Random Forest, LDA, XGBoost, and NaiveBayes were integrated. Through the integration of machine learning techniques, a predictive model for endometriosis was constructed by integrating the machine learning techniques, with the optimal model being determined based on the AUC values derived from various algorithms. Description of all methods used in this study is attached in Supplementary Material S1 with vital parameters to adjust the model for each algorithm.

### 2.3 Diagnostic gene selection

Differential analysis was performed on the GSE51981 dataset, followed by further refinement of the different expressed genes. From 11 distinct machine learning algorithms, diverse combinations of predictive model ensembles tailored for endometriosis were constructed. Subsequently, the optimal predictive model was applied to select the characteristic endometriosis genes from the Differentially Expressed Genes (DGEs) in GSE51981. Nine machine learning algorithms, including Random Forest (RF), Support Vector Machine (SVM), Gradient Boosting Machine (GBM), Least Absolute Shrinkage and Selection Operator (LASSO), eXtreme Gradient Boosting (XGB), Neural Network (NNET), Generalized Linear Model (GLM), k-Nearest Neighbors (KNN) and Decision Tree (DT), were used to evaluate the significance scores and determine the diagnostic genes for each algorithm. Description of all methods used in this study is attached in Supplementary Material S1 with vital parameters to adjust the model for each algorithm.

### 2.4 Validation of diagnostic genes

The ROC curves and AUC values were used to assess the effectiveness and accuracy of the model constructed by the selected genes. The Calibration Curve Plots were used to evaluate the accuracy of the nomogram. The best predictive value is represented by the 45-degree line. The closeness of the curve to perfection determines the accuracy of the results. The clinical utility of the model was evaluated using Decision Curve Analysis (DCA).

### 2.5 Functional enrichment analysis

The Disease Ontology (DO), Gene Ontology (GO), and KEGG pathway enrichment analysis were conducted by using R packages including ClusterProfiler, org. Hs.e.db, Digure, and EnrichPlot. Gene Set Enrichment Analysis (GSEA) was employed to identify significant functional terms between EMS and control samples, plus a GMT reference gene set. Significant enriched terms were determined with a threshold of *p* < 0.025, a q-value of 0.05, and a false discovery rate <0.25 (Yu et al., 2012).

### 2.6 Assessment of immune cell subtype distribution

Following the revelation of the infiltration status of 22 immune cell subpopulations, the CIBERSORTX algorithm was used to investigate the disparities between endometriosis patients and healthy controls regarding *in situ* endometrium (Newman et al., 2019). CIBERSORTX is a computational method based on gene expression data that can estimate the relative abundance of different cell types in a sample. CIBERSORTX was chosen because it provides a more accurate and reliable cell composition analysis, which helps to better understand the cellular heterogeneity of the sample. Furthermore, the abundance of the 22 immune cell subpopulations and the percentage of immune cells were visualized within each sample.

### 2.7 Immune correlation analysis and validation

Spearman’s rank correlation analysis in R was employed to investigate the correlation between identified gene biomarkers and levels of infiltrating immune cells. The visualization of established associations was accomplished using the ggplot2 package. The filtering criterion was set at *p* < 0.05. Furthermore, the findings were validated on a validation dataset for the selected genes. The outcomes were also visualized for better representation.

### 2.8 Determination of combined biomarkers

To select the optimal combination of biomarkers, the diagnostic genes selected by the best algorithm were randomly combined, determining the best predictive combination for each gene count.

## 3 Results

### 3.1 Diagnostic gene selection

**FIGURE 2:**
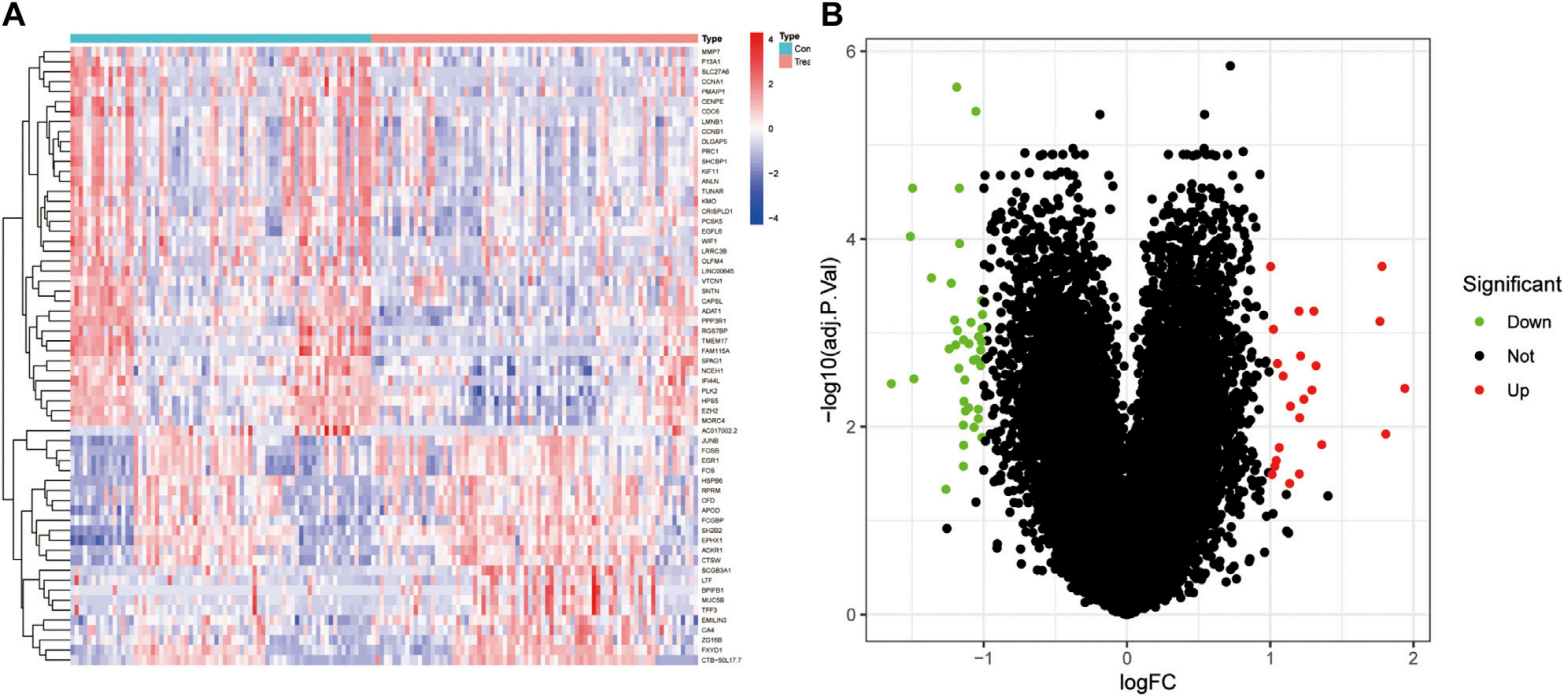
DEGs between EU and EC in the training set. **(A)** Heatmap of DEGs, with red indicating high expression and blue indicating low expression. **(B)** Volcano plot of DEGs, with red indicating upregulation and green indicating downregulation.

**FIGURE 3:**
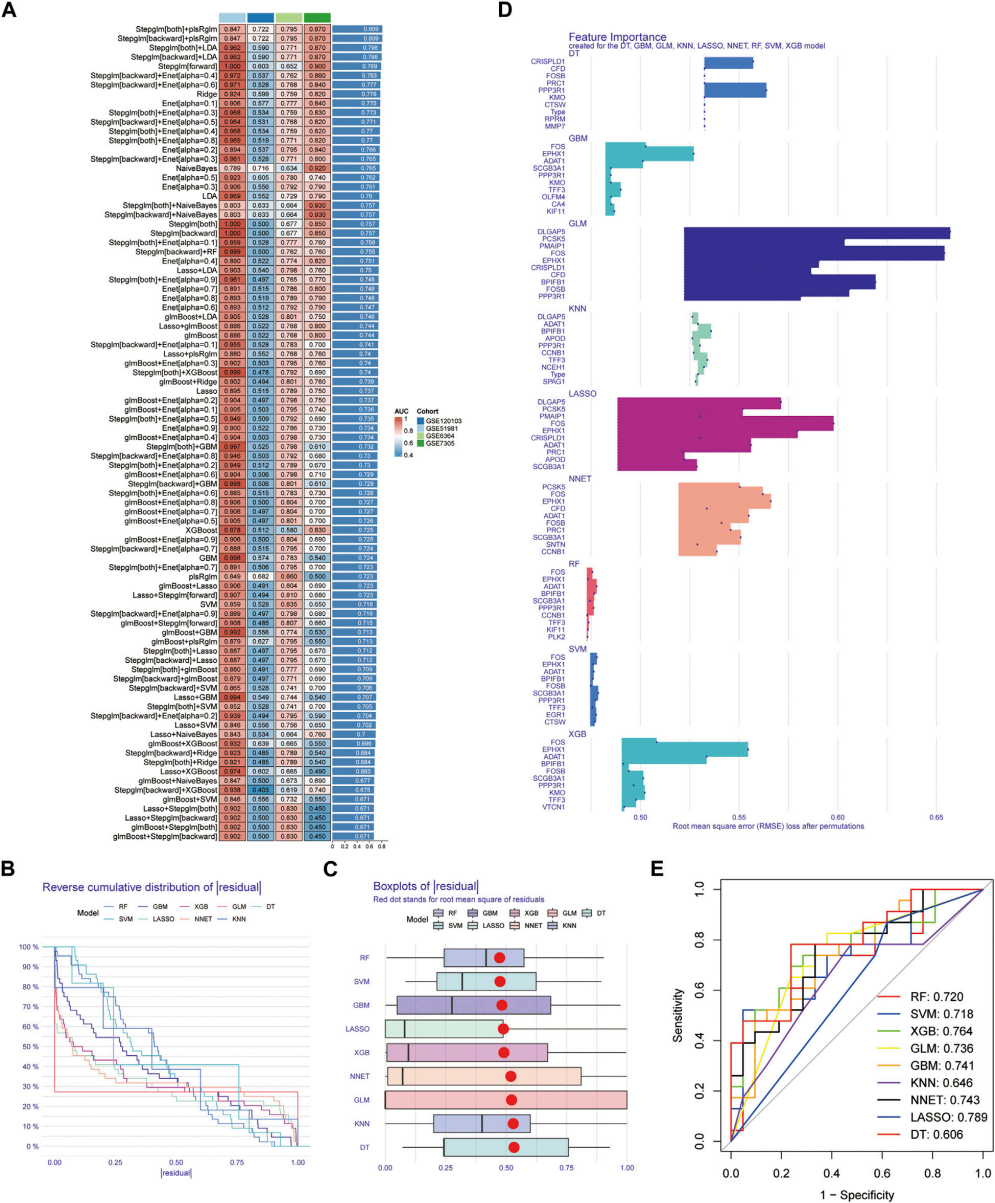
Construction of the predictive model and selection of diagnostic genes. **(A)** AUC values of 113 machine learning algorithm combinations across four testing cohorts. **(B)** Reverse cumulative distribution of residual for diagnostic gene selection using 9 machine learning algorithms. **(C)** Boxplots of residual for diagnostic gene selection using 9 machine learning algorithms, with the red dot representing the root mean square of residuals. **(D)** Feature importance plots created for the DT, GBM, GLM, KNN, LASSO, NNET, RF, SVM, and XGB models. **(E)** ROC curves for diagnostic gene selection using 9 machine learning algorithms.

After conducting differential analysis on the GSE51981 dataset, a total of 62 differentially expressed genes were identified. This analysis was accompanied by visualizations of a heatmap (Figure 2A) and a volcano plot (Figure 2B). Subsequently, 113 predictive models were built using machine learning integration methods. A combination of Stepglm [both] + plsRglm algorithms was selected based on the predictive model’s AUC values to identify 30 characteristic genes (Figure 3A). Then, using nine different algorithms, the diagnostic genes of each algorithm were calculated (Figures 3B–E). Based on the ROC curve and other model evaluation indicators, it was decided to select the top five genes selected by the LASSO algorithm, comprising FOS, EPHX1, DLGAP5, PCSK5 and ADAT1.

### 3.2 Validate diagnostic genes

**FIGURE 4:**
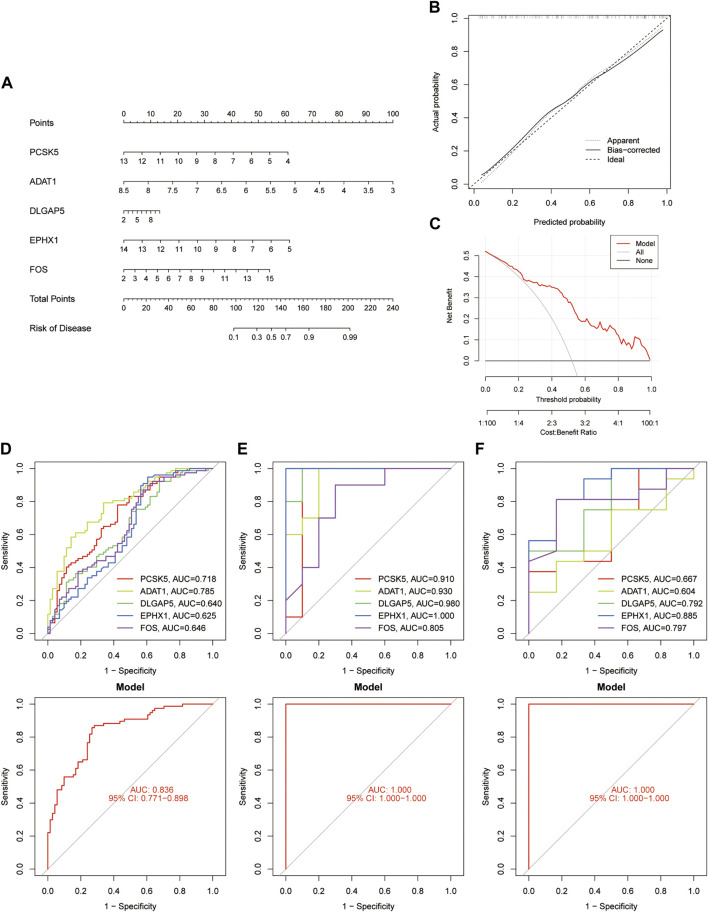
Validation of diagnostic Genes. **(A)** Nomogram depicting validation genes. **(B)** Calibration curve plots illustrating the nomogram. **(C)** Decision Curve Analysis conducted for validation genes. **(D–F)** ROC curve plots depicting feature genes in the GSE51981, GSE7305, and GSE25628 datasets, correspondingly.

A nomogram (Figure 4A) and the calibration curve diagram (Figure 4B) evaluated its accuracy. The clinical utility of the nomogram was evaluated through Decision Curve Analysis (DCA) (Figure 4C). ROC curves were plotted in the validation set, and the high AUC values indicated the model’s predictive capability (Figures 4D–F **).**

### 3.3 Functional enrichment analysis

**FIGURE 5:**
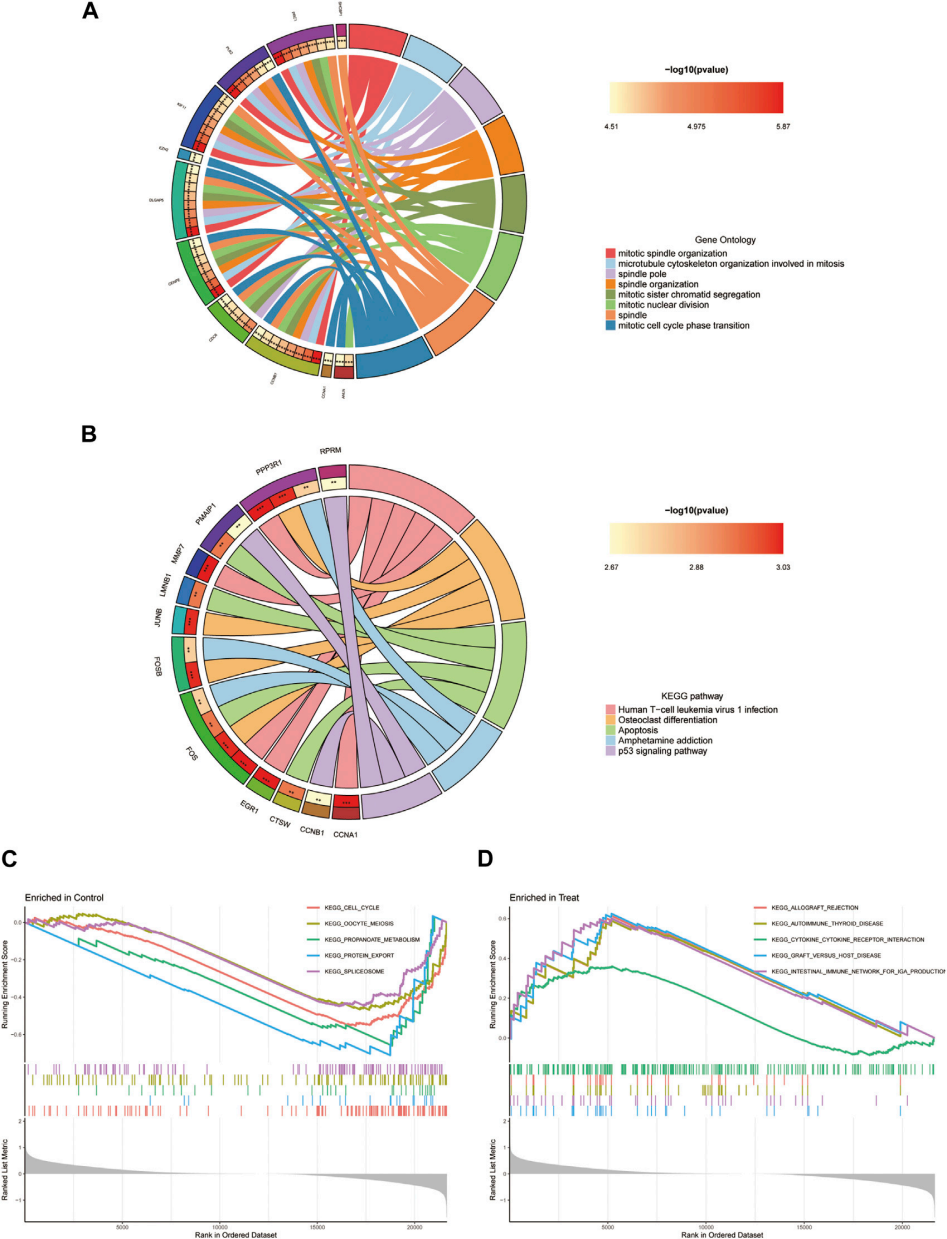
Enrichment analysis of DEGs. **(A)** GO analysis. **(B)** KEGG analysis. **(C, D)** Disease group and health group GSEA analysis.

The results of the GO analysis indicate that the differentially expressed genes are prominently enriched with terms associated with “mitotic spindle organization,” “spindle pole,” “intercellular bridge,” and “serine hydrolase activity” (Figure 5A). Furthermore, the KEGG analysis suggests a potential connection between these differentially expressed genes linked to endometriosis and signalling pathways such as “Human T-cell leukemia virus one infection,” “Osteoclast differentiation,” and “Apoptosis” (Figure 5B). Given the discernible variations in gene expression patterns between the disease and healthy groups, the Gene Set Enrichment Analysis (GSEA) was employed to probe into biological pathways relevant to their distinctive characteristics. The findings strongly point to the disease group’s primary association with pathways such as “ALLOGRAFT_REJECTION,” “AUTOIMMUNE_THYROID_DISEASE,” “CYTOKINE_CYTOKINE_RECEPTOR_INTERACTION,” “GRAFT_VERSUS_HOST_DISEASE,” and “INTESTINAL_IMMUNE_NETWORK_FOR_IGA_PRODUCTION” (Figure 5C). Conversely, the healthy group exhibits connections to pathways such as “CELL_CYCLE,” “OOCYTE_MEIOSIS,” “PROPANOATE_METABOLISM,” “PROTEIN_EXPORT,” and “SPLICEOSOME” (Figure 5D).

### 3.4 Immune infiltration analysis

**FIGURE 6:**
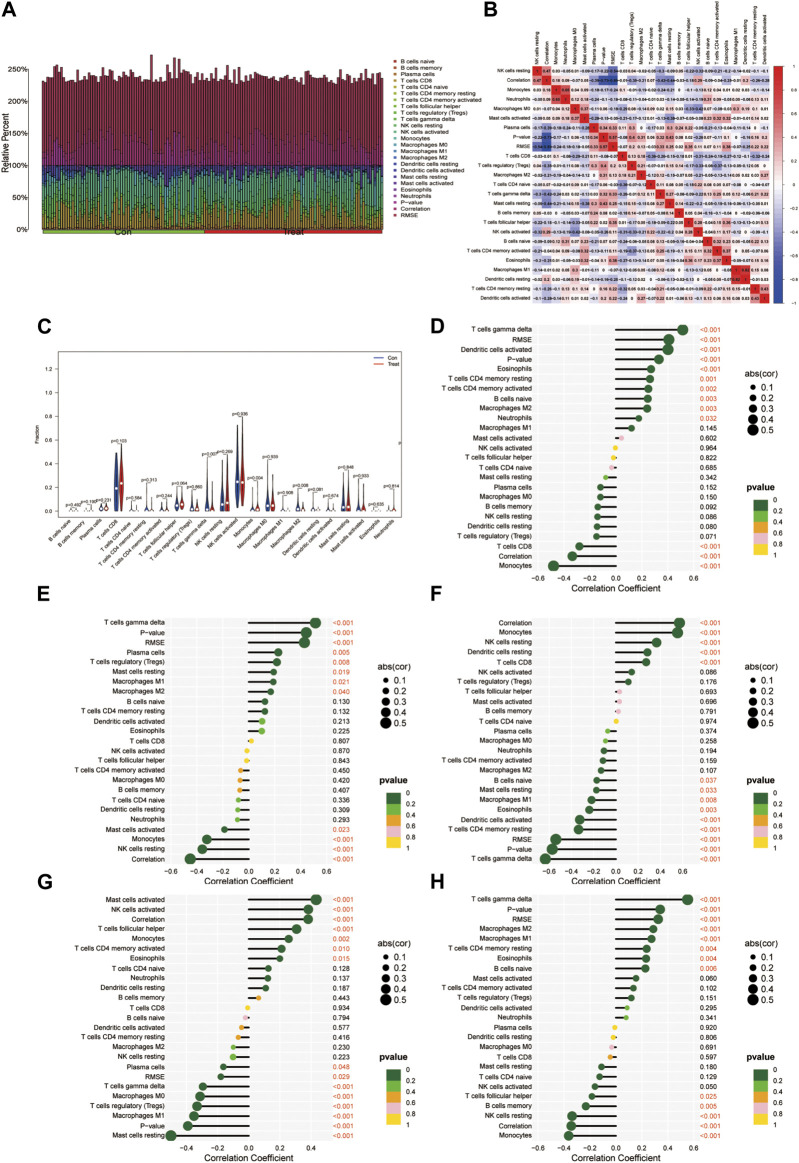
Immunocyte infiltration analysis. **(A)** This figure depicts the degree of infiltration of different immune cells between the diseases and normal groups. **(B)** Immune cell correlation analysis. The horizontal and vertical axes represent the names of immune cells, and the values indicate the correlation coefficients between immune cells. Red indicates positive correlation, while blue indicates negative correlation. **(C)** Violin plots illustrating the differences in immune-infiltrating cells between the diseases group and the normal group. The horizontal axis represents the names of immune cells, and the vertical axis represents the content of immune cells. Blue represents the normal group, and red represents the diseases group. *p* < 0.05 indicates significant differences. **(D-H)**. Correlation analysis of genes ADAT1, PCSK5, DLGAP5, EPHX1, and FOS with immune-infiltrating cells.

The analysis of immune infiltration revealed a slight shift in the immune microenvironment between the disease group and the healthy group (Figures 6A, B). Notable variations were observed in the expression of T cell gamma delta, Monocytes, and Macrophages M2 between these two groups (Figure 6C). The correlation analysis highlighted that the ADAT1 gene displayed a positive correlation with T cell gamma delta, dendritic cells activated, Eosinophils and T cell CD4 memory resting while exhibiting a negative correlation with Monocytes and T cell CD8 (Figure 6D). Similarly, the DLGAP5 gene exhibited a positive correlation with T cell gamma delta, Plasma cells, T cell regulatory (Tregs), and Mast cells resting but showed a negative correlation with NK cells resting, Monocytes, and Mast cells activated (Figure 6E). The gene EPHX1 displayed a positive correlation with Monocytes, NK cells resting, dendritic cells resting, and T cell CD8, while revealing a negative correlation with T cell gamma delta, T cell CD4 memory resting, dendritic cells activated, Eosinophils, and Macrophages M1 (Figure 6F).

Furthermore, the FOS gene demonstrated a positive correlation with Mast cells activated, NK cells activated, T cell follicular helper, Monocytes, and T cell CD4 memory activated, while indicating a negative correlation with Mast cells resting, Macrophages M1, T cell regulatory (Tregs), Macrophages M0, and T cells gamma delta (Figure 6G). Lastly, the gene PCSK5 displayed a positive correlation with T cells gamma delta, Macrophages M2, Macrophages M1, T cells CD4 memory resting, and Eosinophils while revealing a negative correlation with Monocytes, NK cells resting, B cell memory, and T cell follicular helper (Figure 6H).

### 3.5 External validation set validation

**FIGURE 7:**
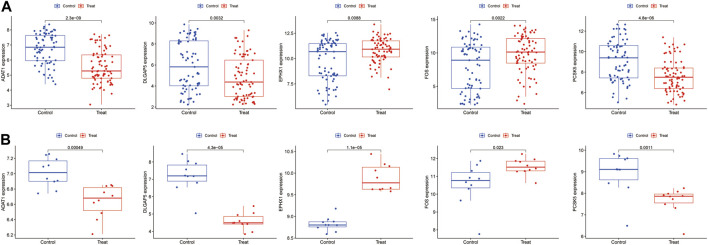
The validation of the five feature genes associated with endometriosis was performed using the datasets **(A)** GSE51918. **(B)** GSE7305.

The validation of the final set of five feature genes was conducted across two datasets: GSE7305 and GSE51981. The results illustrated that the ADAT1, EPHX1, FOS, PCSK5 and DLGAP5 gene were all validated (Figures 7A, B). ADAT1, DLGAP5 and PCSK5 are low expressed genes and EPHX1, FOS are high expressed genes.

### 3.6 Determination of the optimal predictive combination

**FIGURE 8:**
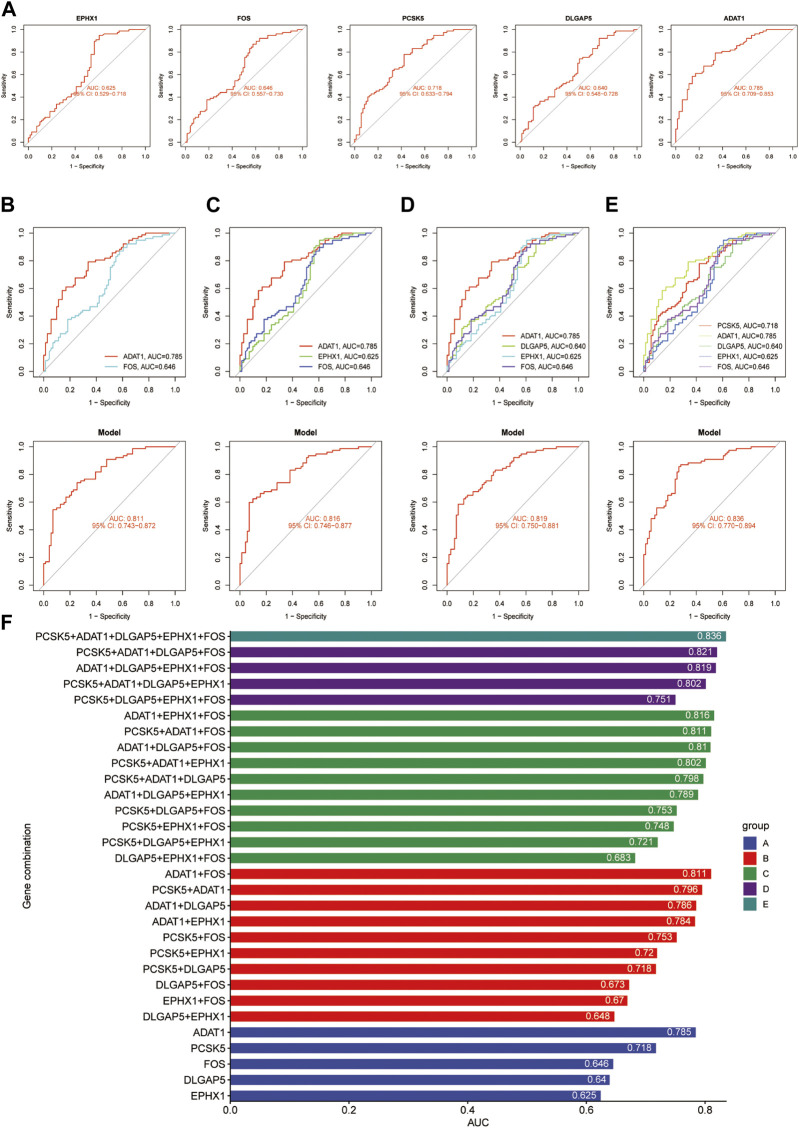
An overview of the optimal combination of genes in the test group. **(A)** ACU values for ADAT1, PCSK5, DLGAP5, EPHX1 and FOS. **(B)** The best combination of two genes to diagnose a gene. **(C)** The best combination of three genes to diagnose a gene. **(D)** The best combination of four genes to diagnose a gene. **(E)** The best combination of five genes to diagnose a gene. **(F)** The AUC values for each gene combination in the test group.

When evaluating the predictive performance of individual genes, the ADAT1 gene demonstrated the best performance, showing a significant predictive effect with an AUC score of 0.785 (Figures 8A, F). In cases involving pairs of genes, the combined impact of the ADAT1 and FOS genes exhibited the most robust performance, achieving an AUC value of 0.811 (Figures 8B, F). Within combinations of three genes, the fusion of ADAT1, EPHX1, and FOS genes displayed the most favourable outcomes, achieving an AUC of 0.816 (Figures 8C, F). For combinations involving four genes, the collaborative effects of the ADAT1, PCSK5, DLGAP5, and FOS genes showcased the highest performance level, resulting in an AUC of 0.821 (Figures 8D, F). Furthermore, the predictive impact of gene combinations involving five members reached an impressive 0.836. The combination of five genes exhibited optimal predictive performance (Figures 8E, F) (Table 2).

**TABLE 2:** An overview of the optimal combination of genes in the test group (five genes).

Gene set	Genes	AUC	Model AUC	Description
1	PCSK5	0.718	0.836 95% CI: 0.770–0.894	Subtilisin/kexin-like protease PC7
	ADAT1	0.785		Adenosine Deaminase
	DLGAP5	0.640		postsynaptic density-95-Associated Protein 5
	EPHX1	0.625		associated with metabolic processes within the body
	FOS	0.646		FBJ osteosarcoma virus (OSV-40) replicon

## 4 Discussion

Endometriosis is a common gynecological disease that affects women of childbearing age. Its causes are complex, and the pathogenic mechanism is unclear. Traditional diagnostic methods rely on surgical exploration and histopathological diagnosis, but these methods are traumatic, high-risk, and costly. At present, most research is limited to single diagnostic biomarkers and has not integrated multiple different biomarkers. In order to provide more comprehensive diagnostic information and improve diagnostic accuracy, machine learning integration methods are used in this study to enhance the performance of predictive models by utilizing multiple algorithms. Using biomarker combination as a new method for diagnosing endometriosis, has significant clinical promise. Predictive models consider the performance of various algorithms, and the accuracy, stability, and other indicators of the model. A predictive model of endometriosis was built using machine learning integration, and the model Stepglm [both] plus the plsRglm algorithm formed the optimal model based on the high and low levels of the AUC.

Nine ML machine learning algorithms were then used to screen for five potential endometriosis diagnostic genes, and the five diagnostic genes were finalized after cross-validation using AUC and other measurements. In order to select the best biomarker combinations for predicting endometriosis diagnosis, the five genes obtained by the LASSO algorithm were randomly combined with one to five genes and the optimal prediction combinations of individual gene numbers were screened. It was found that in the predictive performance comparison of a single gene, ADAT1, the predictive effect was best, with an AUC of 0.785. The prediction for 5 gene combinations (FOS, EPHX1, DLGAP5, PCSK5, and ADAT1) was 0.836. In addition, the diagnostic genes of endometriosis and the characteristics of immune cell infiltration were analyzed. It was found that the selected diagnostic gene was closely related to the infiltration of immune cells.

By analyzing the identified combination biomarkers, this study has gained a deeper understanding of the underlying biological processes associated with endometriosis. The study reveals that these biomarkers involved the cell cycle’s mitosis and cell nucleus division processes. Some related studies have also been reported. For instance, Mori et al. (2015) indicate that the agonist G-1 of G protein-coupled estrogen receptor 1 (GPER-1) induces cell cycle arrest and accumulation in the sub-G phase, leading to apoptosis in endometriosis cells during mitosis. It was also found that cell cycle proteins B1 and Plk1 may also mediate the proliferation of ectopic endometrial cells under the regulation of ovarian hormones (Tang et al., 2009).

Also, the results of KEGG analysis highlight the significant roles played by pathways such as human T-cell leukemia virus 1 infection and the p53 signalling pathway in this process. In severe/advanced endometriosis, anomalies frequently arise in chromosome 17 as a whole, particularly at the p53 locus. This may involve somatic mutations, and the clonal evolution might depend not only on p53 somatic mutations but also on alterations in other oncogenes or tumour suppressor genes (Bischoff et al., 2002). Furthermore, the downregulation of p53 was associated with the risk of Indian women developing endometriosis (Govatati et al., 2012).

FOS1, EPHX1, DLGAP5, PCSK5, and ADAT constitute the optimal set of identifying biomarkers among the identified combination gene set in this study. Numerous studies based on cell or animal models have confirmed that FOS1, EPHX1, and PCSK5 have significant effects on the occurrence and development of endometriosis, which is consistent with the results of this study. We have summarized the impact of these three genes on endometriosis by reviewing existing literature. Previous research has not studied the impact of DLGAP5 and ADAT on endometriosis. This study is the first to report on these two genes, and the potential impacts of these two genes has been summarized.

The FOS gene family comprises a group of gene encoding transcription factors, with the most prominent member being the c-FOS gene. In an open, prospective, and controlled study, it was found that the expression levels of the c-FOS gene were higher in patients with endometriosis compared to normal endometrium. Additionally, immune histochemical results revealed a more abundant distribution of c-FOS protein in the extracellular matrix of endometriosis tissues (Morsch et al., 2009; Reis et al., 2009). Pan et al. (2008) pointed out that the expression of c-Fos in human endometrium might be regulated by estrogen 17β-E, and c-FOS could enhance the development of endometriosis by promoting the expression of the MMP-9 gene, thereby increasing the invasive potential of endometriotic implants. In a study involving the population of Eastern India, the presence of IL-1β induced the phosphorylation of c-FOS protein, further enhancing gene transcription, promoting the production of MMP-13, and increasing the risk of endometriosis (Pandit et al., 2022). This series of research findings encompass multiple crucial roles of c-FOS in developing endometriosis, spanning from gene expression to protein activity. These findings provide a more comprehensive understanding of its role in the disease mechanism.

PCSK5 (Proprotein Convertase Subtilisin/Kexin Type 5) is a protein precursor convertase primarily responsible for processing and activating various cell factors and adhesion factors. These include matrix metalloproteinases, N-cadherin, and insulin-like growth factors, which play pivotal roles in cell signalling, cell adhesion, growth, and differentiation processes. Aberrant activation of these factors may be associated with various diseases, including endometriosis (Sounni et al., 2002; Bassi et al., 2005; Maret et al., 2012).

EPHX1 (Epoxide Hydrolase 1) is a gene that encodes an enzyme belonging to the esterase family, which regulates the metabolism of various compounds inside and outside the organism. The EPHX1 enzyme is primarily involved in the hydrolytic metabolism of epoxides, converting them into corresponding diol compounds, thereby regulating the activity and stability of these compounds (Václavíková et al., 2015). It has been demonstrated that influencing EPHX1 activity or causing its dysregulation may lead to the occurrence of gynecological disorders such as pre-eclampsia, cervical cancer, and ovarian cancer. It is also suspected to be involved in fetal valproate syndrome and diphenylhydantoin toxicity (Yang et al., 2014; Václavíková et al., 2015). In an IVF/ICSI program involving women undergoing infertility treatment, serum vitamin K levels were found to predict embryo quality.

Furthermore, the polymorphism of the EPHX1 gene was found to significantly impact oocyte quality and pregnancy chances, making it a predictive criterion for assessing embryo quality (Khechumyan et al., 2018). The function of the EPHX1 gene might be related to the pathogenesis of endometriosis. The enzyme encoded by EPHX1 participates in metabolizing endogenous and exogenous substances, including hormones, lipids, and environmental pollutants. Studies have revealed that certain genetic variations associated with endometriosis could influence the expression or enzymatic activity of EPHX1, thereby affecting the balance of estrogen metabolism and inflammatory responses. An imbalance in estrogen metabolism and inflammatory responses could lead to abnormal growth of endometriotic tissue and exacerbation of inflammatory responses, ultimately promoting the development and progression of the disease (Cheong et al., 2009; Naidoo et al., 2016; Zhou et al., 2019; van Hoesel et al., 2021).

The “Discs Large Homolog-Associated Protein 5,” (DLGAP5), belongs to the DLGAP family and is encoded on human chromosome 14q22.3. The protein encoded by this gene plays a critical role in cell division, assembly of the mitotic spindle during mitosis, and the formation of kinetochore fibres (K fibres) (Schneider et al., 2017). DLGAP5 is considered an adverse prognostic biomarker in certain diseases, particularly cancer. Its aberrant expression may lead to abnormal cell division, promoting tumour growth and metastasis (Shen et al., 2023; Tang et al., 2023). In the context of endometriosis research, DLGAP5 may be involved in cellular proliferation and differentiation processes, further influencing the development and progression of the disease. However, the precise connection between DLGAP5 and endometriosis has not been fully elucidated to date, and the specific molecular mechanisms require further research.

ADAT1 (Adenosine Deaminase tRNA-Specific 1), participates in nucleic acid metabolism and protein synthesis processes. Current research indicates no direct or known association between ADAT1 and endometriosis. Genetic factors may play a role in the occurrence of endometriosis. Therefore, researchers can investigate whether gene variations or mutations associated with endometriosis involve ADAT1 or related pathways. It is important to emphasize that current literature has not reached definite conclusions about the relationship between ADAT1 and endometriosis. Further experimental evidence and clinical studies are necessary to fully understand the potential role of ADAT1 in endometriosis.

These research findings provide crucial insights for a deeper understanding of the pathogenesis of endometriosis and the development of related treatment strategies. Collectively, the investigations into genes such as ADAT1, PCSK5, DLGAP5, EPHX1, and c-FOS shed light on the molecular mechanisms underlying this disorder and offer valuable information. Furthermore, other potential vital genes have been identified, for instance, rare variations in MMP7 are significantly enriched in ovarian endometriosis and closely associated with specific clinical features, as first discovered by Liu et al. (2022). Jiang et al. introduced LGALS2 and EGR1 as potential new targets for risk prediction and non-invasive diagnosis of endometriosis, providing the potential for personalized medical treatment of EM patients (Jiang et al., 2023). Additionally, TFF3 has been confirmed as a high-risk gene for endometriosis. Recent research indicates that the TFF3-PAR2 inhibitor peptide effectively reduces TFF3 activity, thereby inhibiting the occurrence and progression of endometriosis. This innovative approach brings new hope for managing endometriosis (de Curcio et al., 2018). These observed outcomes are consistent with the endometriosis-associated genes identified in this study, which may also serve as biomarkers for endometriosis.

The immune infiltration analysis results indicate that there are alterations in the immune microenvironment between the disease group and the healthy group. The two groups have specific differences in the expression of T cell gamma delta, Monocytes, and Macrophages M2. Correlation analysis reveals a close association between diagnostic genes and immune-infiltrating cells, consistent with the results of (Braun et al., 1996; Li et al., 2021). Hence, the above five diagnostic genes have the potential to serve as biomarkers for predicting immune therapy responses, enabling personalized treatment to avoid undue burdens on patients through inappropriate treatments. Finally, a list was compiled of genes related to diagnostic genes associated with completed clinical trials using the ClinicalTrials.gov website (https://clinicaltrials.gov/), which serves as a comprehensive database for clinical trial information (Supplementary Table S2).

Currently, the study of diagnostic biomarkers for endometriosis has not yet fully matured, and single markers such as AXIN1, CA-125, and CA-199 have become the focus of research in this area and have shown considerable diagnostic potential. However, in the dataset used in this study, the expression of CA-125 and CA-199 did not show statistical significance. The AUC value of the subject operating characteristic curve (ROC) for AXIN1 was only 0.646, indicating some diagnostic efficacy. The diagnostic ROC value of this study based on the combination of FOS1, EPHX1, DLGAP5, PCSK5, and ADAT biomarkers was 0.836. These findings further emphasize that single markers may not be sufficient to provide adequate diagnostic accuracy in diagnostic marker studies of endometriosis, and that analysis of combinations of markers may be more reliable.

The findings of this study contribute significant support for the early diagnosis and treatment of endometriosis. By leveraging a combination of various machine learning methods, potential biomarkers were uncovered within extensive gene data, opening new avenues for a deeper understanding of the disease’s pathogenesis and the provision of personalized medical strategies. However, due to limited data sources, the generalizability of these results across different populations and clinical scenarios requires further work. While the study results offer novel insights into the early prediction, diagnosis, and treatment of endometriosis, their application in clinical practice necessitates broader clinical trials and validation to ensure their practical feasibility and efficacy.

## 5 Conclusion

In summary, this study extensively investigated the predictive diagnosis of endometriosis, using a machine learning integration approach. It was found that the combination of FOS, EPHX1, DLGAP5, PCSK5, and ADAT1 biomarkers holds substantial diagnostic value. Bioinformatic analysis combined with comprehensive metrics provided a robust foundation for predicting endometriosis. Furthermore, the exploration of immune cell infiltration revealed a close interrelation between five diagnostic genes and the immune system. These findings promise to offer novel avenues for the diagnosis and treatment of endometriosis.

## Data Availability

The raw data supporting the conclusion of this article will be made available by the authors, without undue reservation.
