# Supplementary material for: Machine learning-based integrated identification of predictive combined diagnostic biomarkers for endometriosis
Source: Front Genet. 2023 Nov 27;14:1290036. doi: 10.3389/fgene.2023.1290036 (PMC10720908; doi:10.3389/fgene.2023.1290036)
Supplement: Supplementary file 1 [file Table1.docx]

| **Table 1 Clinical Trials Related to Combined Gene Analysis** | | | | | |
| --- | --- | --- | --- | --- | --- |
| Gene | NCT Number | Conditions | Drugs | Brief Summary | References |
| FOS | NCT00005676 | 1.Cardiovascular Diseases  2.Coronary Disease  3.Coronary Arteriosclerosis  4.1 more | gemfibrozil | To investigate the relative contributions of high density lipoprotein-C (HDL-C) subspecies to risk for coronary heart disease (CHD) in two distinct existing populations (samples from the VA-HIT study and the Framingham Offspring Study [FOS]) as well as the response of these subfractions to gemfibrozil treatment. | (Asztalos, 2004; Asztalos et al., 2002) |
|  | NCT00853034 | Healthy | Dietary Supplement: FOS-IN  Dietary Supplement: AXOS | The purpose of the study is to evaluate the effects of arabinoxylan-oligosaccharides (AXOS) on the colonic metabolism and microbial composition, and to compare these effects with the reference prebiotic fructo-oligosaccharide enriched inulin.. | (Cloetens et al., 2008) |
|  | NCT04534036 | Constipation  Signs and Symptoms  Digestive Signs and Symptoms  1 more | Dietary Supplement: PDS-08  Other: Placebo | The purpose of this study is to investigate the effect of a 9 strain synbiotic consortium comprised of strains with previous pediatric clinical data for use in modulating airway sensitivity, gastrointestinal discomfort, dermatological inflammation, and reduction in the duration and severity of upper respiratory tract infections in a pediatric population. | (Candela, Biagi, Maccaferri, Turroni, & Brigidi, 2012; Jost, Lacroix, Braegger, & Chassard, 2013) |
|  | NCT02518659 | 1.Hypomagnesemia, Intestinal, With Secondary Hypocalcemia  2.Drug Induced Hypomagnesemia | Dietary Supplement: Inulin | This study investigates the application of dietary inulin fibers in users of proton-pump inhibitors with such a hypomagnesemia. | (Hess, Hoenderop, Bindels, & Drenth, 2012; Lameris, Hess, van Kruijsbergen, Hoenderop, & Bindels, 2013) |
|  | NCT04784182 | 1.Anxiety Generalized  2.Breast Cancer Female | probiotic plus prebiotic supplement,placebo | This is a double-blind, placebo-controlled clinical trial in which female breast cancer survivors and/or their female relatives experiencing moderate to severe anxiety symptoms will be randomized to daily consumption of the synbiotic supplement or placebo. | (Smith, Greene, Babu, & Frugé, 2021) |
|  | NCT03573258 | Bariatric Surgery Candidate | Fiber or Placebo | The primary objective is to evaluate the effect of a viscous and fermentable dietary fibre on ad libitum eating in morbidly obese patients before and 6 month after Roux-en-Y gastric bypass (RYGB) surgery. | (Clark & Slavin, 2013; Slavin, 2013) |
| EPHX1 | NCT03486223 | Diabetes Mellitus  Endocrine System Diseases  Glucose Metabolism Disorders  2 more | GSK2256294  Placebo oral capsul | The purpose of this study is to test how soluble epoxide hydrolase (sEH) inhibition with GSK2256294 affects tissue sEH activity and insulin sensitivity. | (Gangadhariah et al., 2017; Luther & Brown, 2016) |

Asztalos, B. F. (2004). High-density lipoprotein metabolism and progression of atherosclerosis: new insights from the HDL Atherosclerosis Treatment Study. *Current opinion in cardiology, 19*(4), 385-391.

Asztalos, B. F., Horvath, K. V., McNamara, J. R., Roheim, P. S., Rubinstein, J. J., & Schaefer, E. J. (2002). Effects of atorvastatin on the HDL subpopulation profile of coronary heart disease patients. *Journal of lipid research, 43*(10), 1701-1707.

Candela, M., Biagi, E., Maccaferri, S., Turroni, S., & Brigidi, P. (2012). Intestinal microbiota is a plastic factor responding to environmental changes. *Trends in microbiology, 20*(8), 385-391.

Clark, M. J., & Slavin, J. L. (2013). The effect of fiber on satiety and food intake: a systematic review. *Journal of the American College of Nutrition, 32*(3), 200-211.

Cloetens, L., De Preter, V., Swennen, K., Broekaert, W. F., Courtin, C. M., Delcour, J. A., . . . Verbeke, K. (2008). Dose-response effect of arabinoxylooligosaccharides on gastrointestinal motility and on colonic bacterial metabolism in healthy volunteers. *Journal of the American College of Nutrition, 27*(4), 512-518.

Gangadhariah, M. H., Dieckmann, B. W., Lantier, L., Kang, L., Wasserman, D. H., Chiusa, M., . . . Gamboa, J. L. (2017). Cytochrome P450 epoxygenase-derived epoxyeicosatrienoic acids contribute to insulin sensitivity in mice and in humans. *Diabetologia, 60*, 1066-1075.

Hess, M., Hoenderop, J., Bindels, R., & Drenth, J. (2012). Systematic review: hypomagnesaemia induced by proton pump inhibition. *Alimentary pharmacology & therapeutics, 36*(5), 405-413.

Jost, T., Lacroix, C., Braegger, C., & Chassard, C. (2013). Assessment of bacterial diversity in breast milk using culture-dependent and culture-independent approaches. *British Journal of Nutrition, 110*(7), 1253-1262.

Lameris, A., Hess, M. W., van Kruijsbergen, I., Hoenderop, J. G., & Bindels, R. J. (2013). Omeprazole enhances the colonic expression of the Mg 2+ transporter TRPM6. *Pflügers Archiv-European Journal of Physiology, 465*, 1613-1620.

Luther, J. M., & Brown, N. J. (2016). Epoxyeicosatrienoic acids and glucose homeostasis in mice and men. *Prostaglandins & other lipid mediators, 125*, 2-7.

Slavin, J. (2013). Fiber and prebiotics: mechanisms and health benefits. *Nutrients, 5*(4), 1417-1435.

Smith, K. S., Greene, M. W., Babu, J. R., & Frugé, A. D. (2021). Psychobiotics as treatment for anxiety, depression, and related symptoms: a systematic review. *Nutritional neuroscience, 24*(12), 963-977.
